# Warming Dwellings

**Published:** 1868-07

**Authors:** 


### Hall's Journal of Health, July, 1868.

### WARMING DWELLINGS.

This is the season for building and altering houses, and as the health and comfort of ourselves and families depend to an important extent on the manner in which dwellings are warmed and ventilated, it is proposed to notice several publications on this subject which represent the most advanced state of knowledge on these interesting and even vital points.

"Anthracite and Health;" 25cts.; A. Williams & Co., 100 Washington Street, Boston, Mass.; a tract of forty pages, being an inquiry into the influence upon health of anthracite coal when used for fuel for warming dwelling houses, with some remarks upon special evaporating apparatus, by George Derby, M.D., Surgeon to the Boston City Hospital, Editor of State registration reports, late Brevet Lieutenant Colonel and Surgeon of United States Volunteers. Every intelligent housekeeper is interested in this most important subject of house warming, and to such Dr. Derby's monogram will be instructive and suggestive. Among the points presented are 1st, that the inconvenience felt by many from furnace heat, and by some from anthracite coal burned in an open grate, is not the result of the dryness of the air, because the saturation point being 100 degrees, sixty is the healthful measure of moisture; but that the adaptability of man to surrounding circumstances is such that he enjoys breathing at zero, and many degrees below it, when the atmosphere has scarcely one degree of moisture. Many invalids revel in the air of Minnesota, the very dryness of which is considered the healthful element, being from five to ten most probably, in the three winter months, that being the measure at Fairfield, Iowa, which is further south and not so cold. On the other hand, New Bedford, Mass., ninety feet above the sea, has a relative humidity of eighty, and is one of the most healthy localities in the United States, there never having been a single case of cholera there, among its citizens. From this Dr. D. argues that the dryness of furnace heat is not chargeable with the discomforts of a furnace heated apartment; nor can it arise from the scorching or burning up of vegetable and animal particles in the air of the room, because the solid substance thus burned would be inappreciable ; but that the tightness about the forehead, oppression, drowsiness, or whatever symptoms differently constituted persons experience, arise from the presence of carbonic oxide gas, that kind which is given out by burning charcoal, and which all know to be deadly ; and science has demonstrated that if even two per cent of this gas is introduced into the common air, an animal breathing it would become insensible in two minutes ; a single breath of carbonic oxide, accidentally breathed by a scientist, caused him to fall to the earth as if pierced by a bullet.

Coal, in burning, gives out three gases, carbonic oxide, carbonic acid and sulphuric acid, also a little vapor of water ; persons have been suffocated by breathing the fumes of a common match, sulphuric acid gas ; and if carbonic acid gas is breathed into the lungs, it will destroy life in a few minutes, not because it is poisonous, but simply because it has no nourishment for the lungs in it ; thus all three of the products of burning anthracite coal are speedily destructive of life, if breathed unmixed ; and even if mixed to the extent of a few per cent with the common air, carbonic oxide is fatal. Dr. D. asserts that if carbonic acid and oxide are mixed and breathed together it is more destructive than either alone. We cannot give up coal as an article of fuel, and a way must be found of getting the heat out of the coal to warm us, and to be breathed into the lungs without the poisonous ingredients referred to. Dr. D. does not suggest it, but we do, to have the furnace in an outhouse to heat water, and let it be sent into the building by iron pipes ; let these pipes run under the floor, thus keeping the floor warm, which is the most important part of a sitting room to be kept warm ; with our present modes of heating, the floor is from ten to twenty degrees colder than the ceiling, and our wives and daughters can testify how much they suffer from cold feet, rendering the whole body uncomfortable, while the mind and temper and disposition are complaining, peevish and fretful. This mode is too expensive for common use. A cheaper method is to have the furnace in the cellar heating the water and sending it through coiled pipes into each room ; this is also expensive, being from one to two thousand dollars for each dwelling. Dr. D. closes his suggestive article by hoping for the time to come that common illuminating gas may be made cheap enough to warm as well as light our dwellings.

"Lectures on Ventilation," a seventy-five cent book ; just published by John Wiley & Son, 2 Clinton Hall, Astor Place, New York, being a course delivered in the Franklin Institute of Philadelphia, by Lewis W. Leeds, special agent of the quarter-master general for the ventilation of government hospitals during the war, and consulting engineer of ventilation and heating for the United States Treasury Department. These lectures are instructively illustrated with cuts and descriptions of experiments ; among the points which will strike the common reader most are, that in winter time the coldest part of a room is the floor ; that a room is warmed first at the ceiling, then on the sides, and as one layer of air gets warm it imparts heat to its neighbor, so that whether by the ordinary located register, or the fire place, the center of a room is the last to be heated. The practical point in Mr. Leed's theory is, that if holes were made in the floor or base board, the bad air would be poured out through them as water is poured out ; and that if warm air was introduced into the apartment at the ceiling, it would come downwards and follow after the heavy bad air which was let out, for the room must be always full oi air, of some kind, and if the bad was let out, fresh pure air would come in, and as it became vitiated, it would also get heavy and follow the other, and thus would a current of air be constantly passing through the room, constituting a proper ventilation. Taking every thing into account, Mr. Leeds inclines to the opinion that the most practical and available method of warming our dwellings at present, is to have hot air, or hot water pipes placed in each room near the windows or doors and let the cold out door air come into the room over and through these coiled pipes, thus being warmed in its passage, it would come in as a warm draught, pure, keeping the floor the warmest part. Since reading Mr. Leeds book we chanced to have a visit from Mr. James W. Ward, connected with the Custom House of this city and who for general intelligence and love of scientific investigtion, experiment and inquiry has few superiors in this country; he stated that, accidentally, and not with any scientific view, one of the rooms in the Custom House has a stack of hot water pipes just inside the door, in front of it; that the door is always open and that the warmth of the floor and the purity of the air in the coldest weather is delightful.

"Ventilation and warming of buildings," illustrated by 54 plates; to which is added a complete description and illustration of the ventilation of Railway Carriages, for both winter and summer, by the Hon. Henry Ruttan, late vice president of the board of Agriculture of Upper Canada; G P. Putnam Publisher. This richly illustrated 8vo. of some 150 pages, is the result of nineteen years of patient experiment and investigation, at an expense of twenty thousand dollars. This system of ventilation is practically presented by B. R. Hawley's "Air Warmer" of which W. A. Pennell & Co. at Normal, Illinois, are general agents for the United States. The distinctive features of Ruttan's system are that pure warmed air ought to come into a room near the ceiling, or over the transom of doors; that the impure, and comparatively colder air should be passed out under the floor into a flue or chimney, thus keeping the floor warm and the whole room ventilated. Theories are comparatively valueless. So we wrote to Pennel & Co. to know if there was any house in New York warmed by their plan; they reply there is not. If they will only come here and let the New Yorkers "see it," they would clear more money in five years than they will in "Normal," in five hundred centuries; where is "Normal?" we thought it was on the Grampion hills, or there away, where somebody's "father fed his flock, a frugal swain!" orperhaps it was "Norvel" we were thinking of. These gentlemen are very enthusiastic and say "there is nothing in the reach of man that would do so much for the health of families as Ruttan's System." To estimate the cost in buildings already erected, they would want to know the size of the rooms, height of ceiling, size and location of all the present flues and a plan of the ground floors. The subject is certainly of great importance ; we have given the titles of the books and of their writers in full, in order to give our readers an idea of the character of the persons who have written ; we have also given the addresses, to save ourselves the trouble of answering letters of inquiry and also to save time to our readers ; especially to those of them who are now building ; the address of Mr. Leeds is 110 Broadway, New York, or Germantown, Penna. Robert Earle Esqr. of New Bedford, one of the most practical ly observant travellers we have ever met with, says that the Russians heat their houses by burning wood in furnaces made of porcelain and that a delightful warmth is maintained all over the building ; that the landlords have to keep the houses heated, thus saving the tenants all trouble ; and that the houses are kept heated whether occupied or not, else all the water pipes would be ruined. One of these furnaces was sent to this country by some enthusiastic United Statesian, but from some cause, it would not work ; besides, wood is too costly. A good many persons on the subject of ventilation, have run mad, stark mad, and have killed themselves as " dead as a door nail " if the reader knows how dead that is, among these last was Dr. Benjamin Franklin, according to the testimony of Thomas Jefferson, author of the Declaration of Independence. Franklin was sick, thought a good deal of the curative virtues of fresh air ; sat by the hour at an open window and died, saying on spilling a part of the last dose, as he was taking it into his trembling hand, "A dying man can do nothing well." The old doctors might say, that if he had taken the whole dose, it would have cured him ; the hydropaths no doubt think that even part of a dose was enough to have killed him, and that if he had taken all, it would have blown him up, or have eaten off his head after he was dead. Many persons, especially ladies, on the instant of taking a seat in a public vehicle, will open the window nearest them, because the air seems " close " ; but " close " as it is, it is not the hundredth part as injurious and fatal even, as a draught of cold air coming in on a person, who to a greater or less extent is tired and over-heated by the previous exercise ; and being so is peculiarly liable to take a cold, to suffer for days and weeks or even longer, while the discomfort of breathing a " close " air, would have been of of but a few moments duration. We do not advise any one to sleep with an open window, if the fire-place and an inner door can be left open ; if this door cannot be left open, then a little fire or a lamp should be kept burning on the hearth all night which would create a current of air up the chimney by which the bad air on the floor would be driven out before the fresh pure air coming in through the crevices of the windows and doors, thus securing a constant current and ventilation.

While the various theories now proposed for warming and ventilating buildings are being sifted and brought to a practical and demonstrably truthful test, it is important to determine what is best to be done under present circumstances, what plans are best and most available to the masses, who occupy houses as they are and have neither permission nor means to alter or make experiments, and must use common coal in stoves, grates, or furnaces ; this is done in the health tract No. 340 on warming houses, in this number ; meanwhile attention is directed to a " Report of the warming and ventilating of the United States Capitol by Professor Henry of the Smithsonian Institution, Washington City, who will send a copy without charge to any one desiring the same ; the conclusion of the report is that the " Capitol " is the best ventilated public building in the world. Wonder if its because our representatives there have such an inveterate habit of ventilating themselves on the floor of Congress ?

